# Phenylalanine: A Promising Inducer of Fruit Resistance to Postharvest Pathogens

**DOI:** 10.3390/foods9050646

**Published:** 2020-05-18

**Authors:** Manish Kumar Patel, Dalia Maurer, Oleg Feygenberg, Amos Ovadia, Yigal Elad, Michal Oren-Shamir, Noam Alkan

**Affiliations:** 1 Department of Postharvest Science of Fresh Produce, Agricultural Research Organization (ARO), Volcani Center, Rishon LeZion 7505101, Israel or manishkp@volcani.agri.gov.il (M.K.P.); daliam@volcani.agri.gov.il (D.M.); fgboleg@volcani.agri.gov.il (O.F.); 2 Agronomia Ltd., Rehovot 76701, Israel; amos@agronomia.co.il; 3 Department of Plant Pathology and Weed Research, ARO, Volcani Center, Rishon LeZion 7505101, Israel; elady@volcani.agri.gov.il; 4 Department of Ornamental Plants and Agricultural Biotechnology, ARO, Volcani Center, Rishon LeZion 7505101, Israel; vhshamir@volcani.agri.gov.il

**Keywords:** phenylalanine, induced resistance, preharvest application, postharvest application, postharvest decay, fungal pathogen

## Abstract

More than 40% of harvested fruit is lost, largely due to decay. In parallel, restrictions on postharvest fungicides call for eco-friendly alternatives. Fruit’s natural resistance depends mainly on flavonoids and anthocyanins—which have antioxidant and antifungal activity—synthesized from the phenylpropanoid pathway with phenylalanine as a precursor. We hypothesized that phenylalanine could induce fruit’s natural defense response and tolerance to fungal pathogens. The postharvest application of phenylalanine to mango and avocado fruit reduced anthracnose and stem-end rot caused by *Colletotrichum gloeosporioides* and *Lasiodiplodia theobromae*, respectively. The postharvest application of phenylalanine to citrus fruit reduced green mold caused by *Penicillium digitatum*. The optimal phenylalanine concentrations for postharvest application were 6 mM for citrus fruits and 8 mM for mangoes and avocadoes. The preharvest application of phenylalanine to strawberries, mangoes, and citrus fruits also reduced postharvest decay. Interestingly, citrus fruit resistance to *P. digitatum* inoculated immediately after phenylalanine application was not improved, whereas inoculation performed 2 days after phenylalanine treatment induced the defense response. Five hours after the treatment, no phenylalanine residue was detected on/in the fruit, probably due to rapid phenylalanine metabolism. Additionally, *in vitro* testing showed no inhibitory effect of phenylalanine on conidial germination. Altogether, we characterized a new inducer of the fruit defense response—phenylalanine. Preharvest or postharvest application to fruit led to the inhibition of fungal pathogen-induced postharvest decay, suggesting that the application of phenylalanine could become an eco-friendly and healthy alternative to fungicides.

## 1. Introduction

More than 40% of the fruit and vegetables harvested worldwide are lost; a large percentage of that loss is related to fungal pathogens that cause postharvest decay [1]. The solution to this issue is complicated due to the restrictions on fungicides that are needed to control those fungal pathogens, calling for inexpensive and safe eco-friendly alternatives to control the postharvest decay of fresh produce [2]. The induction of plant resistance is considered a sustainable strategy to manage the postharvest decay of fruit and vegetables [3,4].

Various biotic or abiotic stresses can trigger plant defenses to create induced resistance [5]. Thus, the induction of the defense response in harvested fruit has the potential to control postharvest disease in an eco-friendly manner [3]. Plant activators are compounds that act on the fruit by triggering defense mechanisms and increasing their tolerance to fungal pathogens [6]. This pathway is known to be triggered in response to various stresses and to play a key role in the induced defense response in plants [7]. The phenylpropanoid pathway is derived from phenylalanine and synthesizes a variety of compounds, such as flavonols, dihydrochalcones, flavones, flavanones, and anthocyanins [8], most of which have both antioxidant and antifungal activity [8,9]. Thus, plants with higher levels of phenolics or flavonoids are more resistant to pathogens [7,10,11,12]. Much research has focused on inducing this pathway to prime the plant defense response [7], or on plant breeding or metabolic engineering for enhancing the quantities of flavonoids [13]. Recently, the amino acid phenylalanine was shown to induce a plant defense response against *Botrytis cinerea* in petunia and tomato leaves [14].

The defense against fungal pathogens in harvested fruit relies on induced and preformed mechanisms [15]. Interestingly, both of these fruit defense mechanisms include the biosynthesis of phenylpropanoids [3,16]. Various inducers, such as jasmonic acid, salicylic acid, riboflavin, harpin, chitosan, and ozone, can increase flavonoid content together with a pleiotropic effect on fruit responses [3,17]. Similarly, biotic and abiotic stresses can induce the phenylpropanoid pathway [5,18]. However, this pleiotropic effect of stress or stress inducers may be accompanied by undesired effects on yield or fruit size, among others. Exogenous priming induces stronger expression of the basal defense response upon pathogen inoculation in treated fruit [19]. Such a priming effect by phenylalanine has never been reported in fruit or in postharvest disease control.

We hypothesized that phenylalanine, a precursor of the phenylpropanoid pathway, would induce a natural defense response in fruit against various fungal pathogens. This amino acid, which is generally regarded as safe (GRAS), could potentially become a practicable application for both pre- and postharvest fruit, due to its relatively low cost and high effectiveness. Optimally, phenylalanine, as an eco-friendly alternative to fungicides, could lead to more marketable fresh produce.

## 2. Methods and Materials

### 2.1. Fungal and Plant Material

Three fungal pathogens were used for this study. *Lasiodiplodia theobromae* strain Avo 62 and *Colletotrichum gloeosporioides* strain Cg-14 were isolated from decayed “Hass” avocado fruit [20,21]. *Alternaria alternata* strain Aa-15 was isolated from rotten mango fruit. All fungi were maintained on potato dextrose agar (PDA, Difco Ltd., Le Pont-de-Claix, France) at 26 °C. To produce conidia, mycelial pieces of *C. gloeosporioides* and *A. alternata* were incubated for 2–4 weeks on a PDA plate at 26 °C. The mycelial pieces of *L. theobromae* were incubated at 26 °C for 20 days on a PDA plate containing a thick layer of autoclaved pine needles [22]. For all fungi, conidia were obtained from the PDA culture plate by flooding the plate with sterile distilled water and filtering through four layers of sterile cheesecloth. The concentration of conidia was adjusted to 5 × 10^5^ conidia/mL. We used four different fruits (mango, avocado, mandarin, and strawberry) to validate this study.

### 2.2. Effect of Phenylalanine on C. gloeosporioides, A. alternata, and L. theobromae Conidial Germination

To evaluate conidial germination, conidia of *C. gloeosporioides*, *A. alternata*, and *L. theobromae* were harvested from 10- to 30-day-old PDA plates. Each fungal sample was diluted to 5 × 10^5^ conidia/mL. The fungal conidial suspension (10 μL) was placed on a glass slide with 8 mM phenylalanine (Sigma, St. Louis, MO, USA) solution (10 μL), and incubated at room temperature in a humid chamber for 20 h, with three biological repeats. The percentage of conidial germination was examined under a light microscope (×40; Leica DM500, Heerbrugg, Switzerland). The averages and SEs of conidia germination percentages were calculated from three biological repetitions for both 8 mM phenylalanine and the water control, and each repeat was counted in three different fields.

### 2.3. Postharvest Treatments

Mango fruits (*Mangiferae indica*) cv. Shelly were harvested from the Sea of Galilee region (northern Israel)—cv. Shelly in August 2019 and cv. Tali in August 2018—and transported to the Agricultural Research Organization (ARO) Volcani Center, Israel. Mango fruits were treated with different concentrations (1, 2, 4, 8, 16, and 32 mM) of phenylalanine dissolved in tap water (neutral pH), by dipping for 30 s, or water as a control and stored at 22 °C for 48 h. The control and treated mango fruits were then inoculated (30 fruits per treatment) with conidia of *C*. *gloeosporioides* on the pericarp (in 1 mm deep, 1 mm diameter wounds with 4 spots per fruit) or of *L*. *theobromae* on the end of a wounded stem, using 7 µL of conidial suspension (per inoculation spot) (5 × 10^5^ conidia/mL); each fruit represented a biological repeat. Then, the inoculated fruits were stored at 22 °C for up to 12 days, and the decayed area was measured periodically until the control fruits were severely decayed.

Mid-season avocado fruits (*Persea americana* cv. Fuerte) were harvested in January 2020 and transported to the ARO Volcani Center, Israel. The avocado fruits were treated with different concentrations (1, 2, 4, 8, 16, and 32 mM) of phenylalanine dissolved in tap water by dipping for 30 s, and the fruit were stored at 22 °C for 48 h. Control and treated avocado fruits were then inoculated (15 fruit per treatment) with conidia of *C*. *gloeosporioides* or *A*. *alternata* on the pericarp (in 1 mm deep, 1 mm diameter wounds with 4 spots per fruit) or of *L*. *theobromae* on the wounded stem end, by placing 7 µL of conidial suspension (per inoculation spot) (5 × 10^5^ conidia/mL); each fruit represented a biological repeat. The inoculated fruits were stored at 22 °C for up to 17 days, and the decayed area was measured periodically until the control fruits were severely decayed.

Mandarin fruits (*Citrus reticulata* cv. Orr) were harvested (March 2019) and transported to the ARO, Volcani Center, Israel. The fruits were treated with different concentrations (2, 6, and 15 mM) of phenylalanine or water as a control (by dipping) for 30 s. The control and treated fruits were stored at 22 °C for 48 h. Control and treated inoculated fruits were sprayed (30 fruits per treatment, each fruit representing a biological repeat) with conidia (5 × 10^5^ conidia/mL) of *P*. *digitatum* and stored, along with control non-inoculated fruits, at 10 °C for 29 days and then moved to 20 °C for 5 days. Decay incidence and severity were measured at 0, 8, 12, 15, 26, 29, and 34 days post inoculation.

Mandarin (*C*. *reticulata* cv. Michal) fruits were harvested and transported to the ARO Volcani Center, Israel. The mandarin fruits were treated with 6 mM phenylalanine for 30 s, and two different infection protocols were used. For early infection, the treated and control fruits were immediately inoculated by spraying with conidia (5 × 10^5^ conidia/mL) of *P*. *digitatum*. For late infection, the treated and control fruits were stored at 22 °C for 48 h then inoculated by *P*. *digitatum* conidial spray (5 × 10^5^ conidia/mL). Each treatment consisted of four 10 kg cases of fruit, each case representing a biological repeat. Both the early- and late-inoculated fruits were stored at 22 °C for 5 days, and decay incidence or severity was measured.

### 2.4. Phenylalanine Concentration in Treated Fruit

Mandarins (*C. reticulata* cv. Michal) and avocadoes *(P. americana* cv. Reed) were dipped for 30 s in 8 mM phenylalanine in cold water (20 °C) or in hot water (50 °C) or embedded in 12% polyethylene wax. After the dipping, the fruits were let to dry. Five hours after the treatments, each fruit was cut in two. Four halves of the mandarin fruits or three halves of the avocado fruits were ground together to obtain one sample of 30 mL. The samples were frozen in liquid nitrogen and lyophilized (three samples, 30 mL for each treatment). Extraction was performed on 20 and 60 mg dry weight (DW) samples of mandarins and avocadoes, respectively, with 3 replicates for each treatment. The GC-MS (GC-7890A, MS-9575C, Agilent Technologies) analysis was done according to Mintz-Oron [23].

### 2.5. Preharvest Treatments

Average-size trees of mango *(M. indica*) cv. Keitt, late in the season (October, 2019) (3 trees per repeat, 4 repeats per treatment), were sprayed with water (control) or with 8 mM phenylalanine on different days (Day 2, 4, or 7, or their combinations) prior to harvest. The control and treated fruits were harvested and transported to the ARO Volcani Center, Israel and immediately stored at 12 °C for 14 days. Some of the control fruits were subjected to postharvest treatment; these were dipped in 8 mM phenylalanine solution for 30 s, stored for 48 h at 22 °C to induce resistance, and then transferred to 12 °C for 14 days. The fruit were then stored for an additional 14 days at 20 °C to mimic long shelf life. Each treatment included four 4 kg cardboard boxes with eight mango fruits; each box was harvested from the middle tree of a randomized block and represented a biological repeat. The natural decay (mainly from *A. alternata* and less from *C. gloeosporioides*) index and percentage were measured after cold storage and shelf life.

Strawberry (*Fragaria ananassa* cvs. Gilli and Malach) bushes were sprayed (10 bushes per repeat, 4 repeats per treatment) with 6 mM phenylalanine in the growing house, 1, 2, or 3 weeks (or their combinations) prior to harvest. After the treatment, fruits were harvested and stored at 5 °C for 12 days. Each treatment consisted of four 500 g plastic boxes, each box considered a biological repeat. Natural decay incidence and severity were measured after 0, 4, 7, and 12 days of storage. Mandarin (cv. Michal) trees (3 trees per repeat, 4 repeats per treatment) were also sprayed in the orchard with 6 mM phenylalanine 3 days prior to harvest. The harvested fruit were transported to the ARO Volcani Center, Israel, stored at 5 °C for 24 days and then moved to 20 °C for 5 days. Each treatment included four 4 kg cardboard boxes, each box representing a biological repeat. The natural decay incidence and severity were measured 0, 7, 10, and 21 days after cold storage (24 days) and after shelf life (29 days).

Postharvest fruit decay in passive inoculated fruit was characterized separately for the incidence (percentage of decayed fruits in a box) and severity of decay (index 0–5; 0—no decay, 1—initial decay, 3—spread decay, 5—wide spread decay). In the wound inoculated fruit, the decay diameter was measured in two different directions and the average diameter was used to calculate the radius; the area was calculated by πr^2^.

### 2.6. Statistical Analysis

Data are presented as mean ± standard error. Statistical significance was determined by either one-way ANOVA (Tukey–Kramer HSD test or Wilcoxon test, for parametric and non-parametric analysis, respectively) or t-tests, using the JMP software (JMP Pro 14 software, SAS Institute, Cary, NC, USA). Different letters or asterisks indicate significant (*p* ≤ 0.05) differences.

## 3. Results

### 3.1. Postharvest Application of Phenylalanine Increases Fruit Resistance to Fungal Pathogens

**Figure 1:**
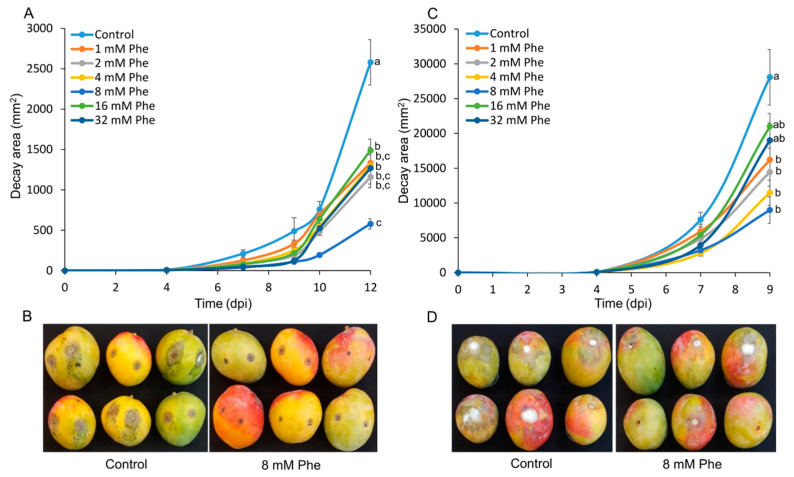
Effect of postharvest treatment with phenylalanine (Phe) on the decay area caused by *C. gloeosporioides* or *L. theobromae* in mango fruit. “Shelly” mango fruits were treated with different concentrations (1, 2, 4, 8, 16, or 32 mM) of Phe or water (control) and, 2 days later, inoculated with either *C. gloeosporioides* or *L. theobromae*. (**A**) Area of anthracnose decay caused by *C. gloeosporioides* during 12 days post inoculation (dpi). (**B**) Representative picture of mango fruits treated with 8 mM Phe and controls, 10 dpi. (**C**) Area of stem-end rot caused by *L. theobromae* during 9 dpi. (**D**) Representative picture of mango fruit treated with 8 mM Phe and controls, 9 dpi. Values are mean ± SE. Different letters indicate a significant difference between treatments according to one-way ANOVA, *p* ≤ 0.05.

Harvested mango “Shelly” fruits were treated with different concentrations of phenylalanine for 30 s and then stored at 22 °C for 48 h to induce resistance. The control and treated fruits were then inoculated with *C. gloeosporioides* conidia. The fruits treated with 8 mM phenylalanine had a significantly smaller decay area than the nontreated controls and the fruits treated with other phenylalanine concentrations (Figure 1a). Similarly, stem-end rot caused by *L*. *theobromae* in mango fruits treated with 8 mM phenylalanine covered a smaller area in comparison to controls and fruits treated with other phenylalanine concentrations (Figure 1c). This experiment was repeated with mango cv. Tali, and similar results were obtained for both pathogens (Figure S1a,c). Representative pictures illustrate the marked differences in both side decay and stem-end rot severity between the nontreated control fruits and those treated with 8 mM phenylalanine (Figure 1b,d, Figure S1b,d). Treatments with increasing concentrations of phenylalanine reduced the decay caused by both *C. gloeosporioides* and *L. theobromae* in a concentration-dependent manner until 8 mM. At higher concentrations, the pathogen-inhibiting effect of phenylalanine decreased (Figure 1a,c, Figure S1).

**Figure 2:**
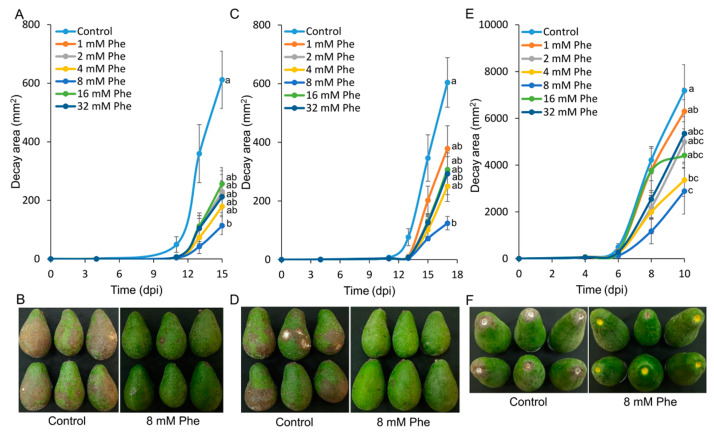
Effect of postharvest treatment with phenylalanine (Phe) on decay caused by *C. gloeosporioides*, *A. alternata*, or *L. theobromae* in avocado fruit. “Fuerte” avocado fruits were treated with different concentrations of Phe (1, 2, 4, 8, 16, or 32 mM) or water (control) and, 2 days later, inoculated with *C. gloeosporioides*, *A. alternata*, or *L. theobromae.* (**A**) Area of anthracnose decay caused by *C. gloeosporioides*. (**B**) Representative picture of avocado fruits treated with 8 mM Phe and controls, 13 days post inoculation (dpi). (**C**) Area of decay caused by *A. alternata.* (**D**) Representative picture of avocado fruits treated with 8 mM Phe and controls, 15 dpi. (**E**) Area of stem-end rot caused by *L. theobromae*. (**F**) Representative picture of avocado fruits treated with 8 mM Phe and controls, 8 dpi. Values are mean ± SE. Different letters indicate a significant difference between treatments according to one-way ANOVA, *p* ≤ 0.05.

Avocado “Fuerte” fruit were treated with different concentrations of phenylalanine for 30 s and stored at 22 °C for 48 h to induce fruit resistance. The fruit were then wound-inoculated with *C*. *gloeosporioides* or *A*. *alternata* conidia on the peel. The decayed area caused by both fungal pathogens on avocado fruits treated with 8 mM phenylalanine was significantly reduced compared to those with other phenylalanine concentrations and the nontreated control fruit (Figure 2a,c). In addition, the stem ends of the treated fruits were inoculated with *L*. *theobromae* conidia. The fruits treated with 8 mM phenylalanine had significantly smaller areas of stem-end rot compared to controls and fruit treated with the other phenylalanine concentrations (Figure 2e). Representative pictures illustrate the marked differences in the severity of side decay caused by *C*. *gloeosporioides* or *A*. *alternata* and the stem-end rot caused by *L*. *theobromae* with the 8 mM phenylalanine treatment compared to the nontreated control (Figure 2b,d,f). In avocadoes, the phenylalanine concentration-dependent effect showed a trend similar to that in mango (Figure 2a,c,e).

Mandarin fruit is known to be extremely vulnerable to *P*. *digitatum*. Mandarin fruits were treated with different concentrations of phenylalanine and stored at 22 °C for 48 h to induce resistance. The fruits were then inoculated with *P*. *digitatum* and stored at a lower temperature. All the concentrations of phenylalanine (2, 6, and 15 mM) reduced decay incidence and severity (Figure S2a,b). The treatment with 6 mM phenylalanine was more effective at inhibiting green mold decay caused by *P*. *digitatum* than the other phenylalanine concentrations or no treatment (Figure S2a,b). The fruit quality (firmness, Brix, and acidity) was not spoiled by the phenylalanine treatments.

### 3.2. Preharvest Application of Phenylalanine Increases Fruit Resistance to Fungal Pathogens

**Figure 3:**
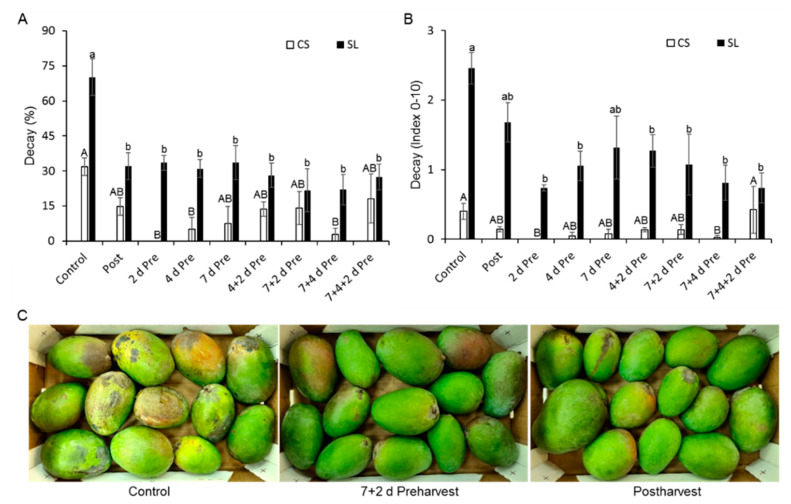
Effect of preharvest phenylalanine treatments on postharvest decay in mango. A “Keitt” mango orchard was sprayed preharvest (Pre) with water or 8 mM phenylalanine 2, 4, or 7 days (d)—or their combination—before harvest, or fruits were treated with 8 mM phenylalanine postharvest (Post). All the fruits were cold-stored (CS) at 12 °C for 14 d and then shelf life (SL)-stored at 22 °C for 10 d. (**A**) Incidence of natural decay (percentage). (**B**) Severity of natural decay (index, 0–10). (**C**) Representative pictures of mango fruits treated with 8 mM phenylalanine preharvest (7 + 2 d preharvest) or postharvest and controls, after shelf-life storage. Values are mean ± SE. Different letters indicate a significant difference between treatments at a specific time point (upper case letters for CS; lower case letters for SL) according to one-way ANOVA, *p* ≤ 0.05.

The preharvest application of phenylalanine was conducted to induce mango, strawberry, and mandarin resistance to fungal pathogens after harvest. Mango “Keitt” fruits were sprayed with 8 mM phenylalanine on different days (2, 4, 7, or their combinations) prior to harvest. Preharvest treatment reduced the postharvest decay in stored mangoes (Figure 3). The preharvest treatments did not spoil the fruit’s quality (firmness, Brix, and acidity). However, overall, all preharvest and postharvest phenylalanine treatments reduced decay (caused by *A. alternata* or *C. gloeosporioides*) (Figure 3a,b). The combination of two preharvest treatments led to an additional, albeit non-significant, reduction in fruit decay (Figure 3a,b). Representative pictures illustrate the marked difference in fruit decay between the nontreated control and the preharvest and postharvest treatments (Figure 3c).

**Figure 4:**
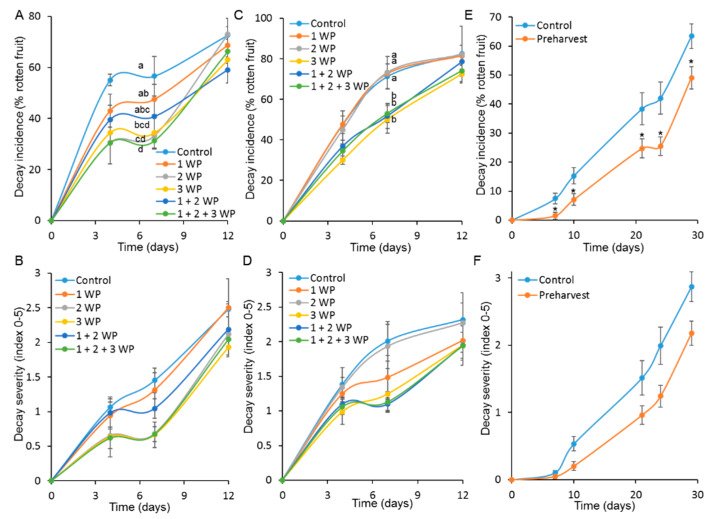
Effect of preharvest application of phenylalanine on postharvest decay in strawberry and mandarin. “Malach” or “Gilli” strawberry plants were sprayed preharvest with water or 6 mM phenylalanine 1, 2, or 3 weeks preharvest (WP) (or their combination). After harvest, the fruits were stored at 10 °C for 12 days. (**A**) Decay incidence (percentage of rotten fruit) and (**B**) decay severity (index, 0–5) in strawberries cv. Gilli. (**C**) Decay incidence and (**D**) decay severity in strawberries cv. Malach. (**E**,**F**) “Michal” mandarin orchard was sprayed with water or 6 mM phenylalanine 3 days before harvest. (**E**) Decay incidence and (**F**) decay severity in harvested fruit. Values are mean ±  SE. Different letters indicate a significant difference (*p ≤* 0.05) according to t-tests or one-way ANOVA.

In strawberries, the preharvest application of 6 mM phenylalanine 1, 2, or 3 weeks (or their combination) prior to harvest reduced postharvest decay (Figure 4). Overall, phenylalanine treatment significantly reduced disease incidence and severity in most of the preharvest treatments. The preharvest application of phenylalanine 3 weeks prior to harvest reduced decay incidence most efficiently, albeit non-significantly, compared to the other preharvest treatments (cvs. Gilli and Malach) (Figure 4a,c), The addition of a second or third preharvest application did not improve the strawberries’ resistance to fungal pathogens (Figure 4a–d). Similarly, 6 mM phenylalanine was applied 3 days before harvest to mandarin cv. Michal fruits to induce resistance to postharvest disease. Indeed, the preharvest treatment with phenylalanine significantly reduced postharvest decay incidence and severity in mandarins compared to the nontreated control (Figure 4e,f).

### 3.3. Mode of Action of Phenylalanine on Conidial Germination and Induced Resistance in Fruit

To examine whether phenylalanine has a direct effect on fungal pathogens, conidia of *C. gloeosporioides*, *A. alternata*, and *L*. *theobromae* were germinated in the presence of 8 mM phenylalanine. The percentage of *L. theobromae* conidial germination was significantly higher when the conidia were exposed for 20 h to phenylalanine compared to the control (94.86% and 88.78%, respectively) (Figure S3). Similarly, the percentage of conidial germination of *C. gloeosporioides* and *A. alternata* was non-significantly higher in the presence of phenylalanine compared to the control (Figure S3). Thus, *C. gloeosporioides*, *L*. *theobromae*, and *A. alternata* germinated better in the presence of phenylalanine, indicating that phenylalanine is not toxic to the fungi.

**Figure 5:**
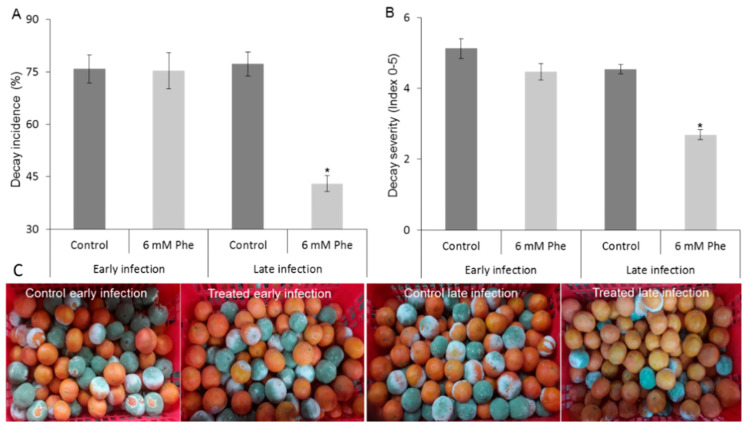
Induced resistance by phenylalanine application in mandarin fruit. “Michal” mandarin fruits were treated with water (Control) or 6 mM phenylalanine. Then, the fruit was either immediately inoculated with *P. digitatum* (early infection) or inoculated 2 days after the phenylalanine application (late infection) and stored at 22 °C. Decay was evaluated 5 days later. (**A**) Decay incidence (percentage of rotten fruit). (**B**) Decay severity (index, 0–5). (**C**) Representative picture of mandarin fruit treated with 6 mM phenylalanine compared to controls, under early- and late-infection protocols. Values are mean ± SE. Asterisk (*) represents a statistically significant difference between the treatment and control according to t-tests *p* ≤ 0.05.

To evaluate whether phenylalanine induces the fruit defense response, mandarin “Michal” fruits were treated with 6 mM phenylalanine just before inoculation with *P. digitatum* (early infection) or 2 days before the inoculation. Phenylalanine treatment did not inhibit green mold when it was applied just before the inoculation with *P. digitatum* (Figure 5). However, when the phenylalanine was applied 2 days before inoculation, it significantly reduced the decay incidence and severity caused by *P. digitatum* (Figure 5a,b). Representative pictures illustrate the marked differences in disease incidence among the different treatments (Figure 5a,c). Thus, it seems that the induction of fruit resistance by phenylalanine needs time to activate the fruit response and inhibit the decay caused by fungal pathogens.

To evaluate if there are residues of phenylalanine in the treated fruit, avocado and mandarin fruits were treated with 8 mM Phe in cold or hot water or embedded in wax. Five hours later, the phenylalanine concentrations in the treated and untreated fruit were similar (Figure S4), suggesting a rapid penetration and metabolism of phenylalanine in the fruit.

## 4. Discussion

Fresh produce losses due to postharvest decay are often caused by fungal pathogens [15,24]. In most cases, the pathogens penetrate the fruit in the field. At that stage, the unripe fruit is resistant and the fungi will enter quiescence until fruit ripening [25]. Many changes occur during fruit ripening, together with a reduction in the natural defense response; this is sensed by the fungi, which switch to their aggressive stage and cause postharvest decay [15]. Postharvest fungicide treatment is the most effective way of controlling the colonization of fungal pathogens and postharvest decay [26]. However, due to the emergence of resistant fungal isolates, toxicity to humans and the environment, and public concern, the use of fungicides has been restricted [3]. Therefore, alternatives are needed.

Phenylalanine is the main precursor of the phenylpropanoid-biosynthesis pathway [7,27]. This pathway plays a major role in fruit’s defense response to fungal pathogens [3,28]. Several elicitors and treatments have been reported to improve host resistance through the induction of the phenylpropanoid pathway [3,29]. Recently, it was found that the phenylalanine treatment of tomato and petunia leaves increases the level of flavonoid compounds and leads to a reduction in the decay caused by *Botrytis cinerea* [14]. In the current study, we found that a single treatment with phenylalanine of harvested fruit 2 days before inoculation with different pathogenic fungi leads to reduced decay in various pathosystems: *C. gloeosporioides* in mango and avocado fruit, *L. theobromae* in mango and avocado fruit, *A. alternata* in avocado fruit, and *P. digitatum* in citrus fruit (Figure 1 and Figure 2, Figures S1 and S2), which supports phenylalanine’s role in inducing resistance in harvested fruit against fungal pathogens. The optimal concentration of phenylalanine for postharvest application was found to be 6 mM for citrus fruits, and 8 mM for mangoes and avocadoes. Up to this optimum, the efficiency of decay control increased with increasing phenylalanine concentration. At an application of 16 mM phenylalanine or higher, a thin white powder appeared on the fruit, indicating that the concentration was probably too high and that the amino acid maybe started to crystalize [30], decreasing the efficiency of postharvest fungal pathogen control. The crystalized phenylalanine is likely not metabolized in the fruit, and the fungal pathogens might use the remaining amino acid as a food source.

Phenylalanine treatment could also be applied preharvest to induce the natural fruit defense response and reduce the decay caused by fungal pathogens. Due to the strawberry’s physiological structure, it is challenging to treat it after harvest with a water-based solution. Therefore, preharvest treatments with phenylalanine were applied to strawberries and showed an inhibition of postharvest decay, probably due to induced resistance (Figure 4). Similarly, the preharvest application (2–7 days preharvest) of phenylalanine to citrus or mango orchards, with a high inoculum rate, significantly reduced the natural postharvest decay as compared to that in control fruit (Figure 3 and Figure 4). Altogether, our results revealed that phenylalanine application either preharvest or postharvest plays a positive role in reducing decay caused by various fungal pathogens in mango, citrus, avocado, and strawberry fruits without negative effects on fruit quality.

To better understand the mode of action of phenylalanine’s inhibition of the decay caused by fungal pathogens, two initial experiments were conducted to evaluate the direct inhibition of the fungal pathogens and induced resistance by phenylalanine. In vitro, the presence of phenylalanine did not suppress the conidial germination of *C. gloeosporioides*, *L. theobromae*, or *A. alternata*, but rather increased it. These results proved that phenylalanine’s mechanism of action in controlling postharvest decay does not involve direct antifungal activity (Figure S3), and suggested that the fungi use phenylalanine as a food source when it is applied without the presence of their host. To evaluate whether phenylalanine acts through induction of the fruit defense response, citrus fruits were inoculated with *P. digitatum* immediately after or 2 days after treatment with phenylalanine. Phenylalanine had no effect when the fruit did not have enough time to acquire natural defenses (Figure 5). However, the application of phenylalanine 48 h before the inoculation led to metabolism of the phenylalanine and the induction of the fruit’s natural defense response, which significantly inhibited postharvest decay (Figure 5). Indeed, five hours after phenylalanine application, there were no residues from the treatment, and all the applied phenylalanine was metabolized (Figure S4). As already noted, phenylalanine is a precursor for the phenylpropanoid pathway [7], and its application to petunia and tomato leaves activates the phenylpropanoid pathway and their resistance to *B. cinerea* [14]. Several secondary metabolites of the phenylpropanoid pathway, such as phenolics, flavonoids (flavonols and iso-flavonoids), and anthocyanins are known compounds that are synthesized by plants, which play crucial roles as signaling molecules in defense mechanisms against pathogens [31,32]. Here, we show that the preharvest or postharvest application of phenylalanine can also induce the fruit’s defense response and improve its resistance to various fungal pathogens.

Similarly, treatments such as jasmonic acid, riboflavin, harpin, chitosan, hot water, ozone, or even various biological control agents can activate the fruit defense response, leading to the induction of the phenylpropanoid pathway and flavonoid synthesis [2,3]. However, those treatments induce a minor abiotic or biotic stress in the fruit and may have a pleiotropic effect on fruit responses, whereas phenylalanine is hypothesized to mainly affect the phenylpropanoid pathway. Phenylalanine has a lower cost in comparison to most chemical and biological treatments. Therefore, we find it to be competitive from an economic point of view. Further research is needed to better understand the mechanism of action underlying the applied phenylalanine’s induction of fruit resistance. Moreover, it should be emphasized that a single treatment with phenylalanine is not as effective as that with a synthetic fungicide, and a combination of various preharvest or postharvest treatments should be studied to design an eco-friendly approach to controlling postharvest disease in each type of fresh produce.

## 5. Conclusions

The current work shows, for the first time, that the preharvest or postharvest application of phenylalanine to various fruits activates the fruits’ defense responses and leads to the inhibition of postharvest decay caused by different fungal pathogens. This novel application of the amino acid phenylalanine could become an eco-friendly and healthy alternative to fungicides.

## Supplementary Materials

The following are available online at https://www.mdpi.com/2304-8158/9/5/646/s1, Figure S1: Effect of postharvest phenylalanine (Phe) treatment on decay caused by *C. gloeosporioides* or *L. theobromae* inoculation of mango fruit, Figure S2: Effect of postharvest treatment with phenylalanine (Phe) on green mold decay caused by *P. digitatum* inoculation in mandarin fruit, Figure S3: Direct effect of phenylalanine on conidial germination, Figure S4: Phenylalanine residue in treated and untreated mandarin and avocado fruit.
