# Supplementary material for: Phenylalanine: A Promising Inducer of Fruit Resistance to Postharvest Pathogens
Source: Foods. 2020 May 18;9(5):646. doi: 10.3390/foods9050646 (PMC7278716; doi:10.3390/foods9050646)
Supplement: Supplementary file 1 [file foods-09-00646-s001.pdf]

Phenylalanine: A Promising Inducer of Fruit Resistance to Postharvest Pathogens

Manish Kumar Patel[1], Dalia Maurer[1], Oleg Feygenberg[1], Amos Ovadia[2], Yigal Elad[3], Michal Oren-Shamir[4] and Noam Alkan[1*]

[1] Department of Postharvest Science of Fresh Produce, Agricultural Research Organization (ARO), Volcani Center, Rishon LeZion 7505101, Israel; manishkp@volcani.agri.gov.il (M.P.K); daliam@volcani.agri.gov.il
(D.M); fgboleg@volcani.agri.gov.il (O.F); noamal@volcani.agri.gov.il (N.A)

[2] Agronomia Ltd., Israel; amos@agronomia.co.il (A.O)

[3] Department of Plant Pathology and Weed Research, ARO, Volcani Center, Rishon LeZion 7505101, Israel; elady@volcani.agri.gov.il (Y.E)

[4] Department of Ornamental Plants and Agricultural Biotechnology, ARO, Volcani Center, Rishon LeZion 7505101, Israel; vhshamir@volcani.agri.gov.il (M.O-S)

* Correspondence: noamal@volcani.agri.gov.il (N.A.)

Supplemental Figures

Figure S1

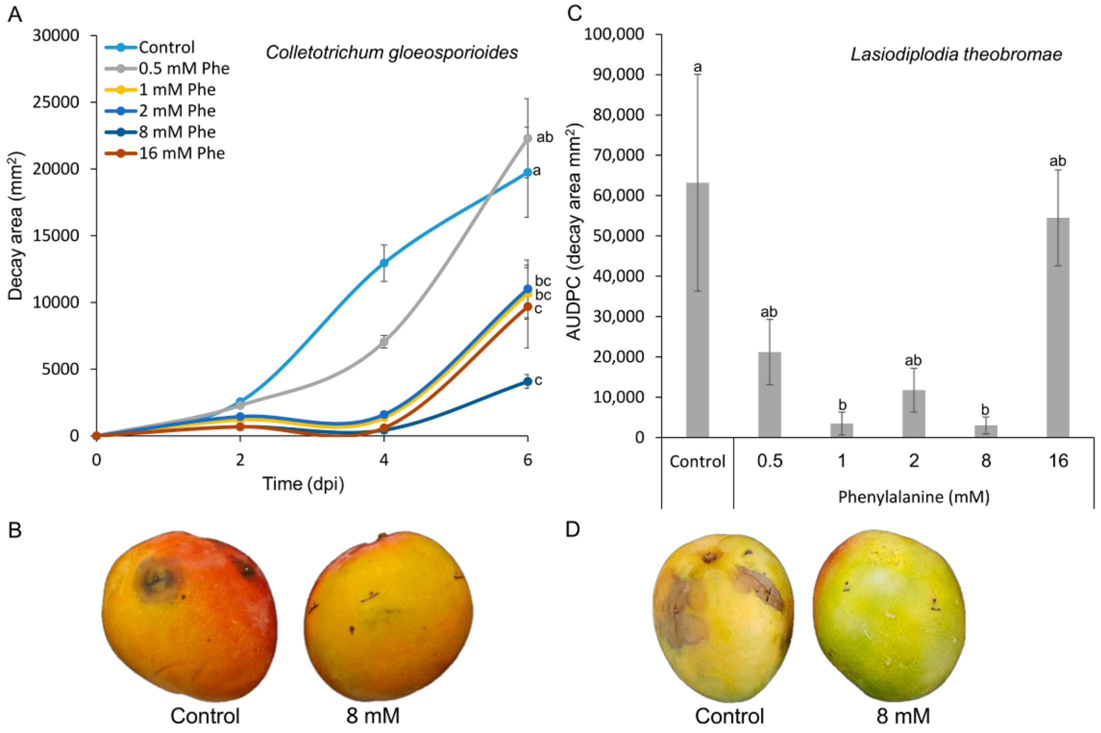

Figure 1. Effect of postharvest phenylalanine (Phe) treatment on decay caused by *C. gloeosporioides* or *L. theobromae* inoculation of mango fruit. 'Tali' mango fruit were treated with different concentrations of Phe or water (control) and incubated at room temperature for 2 days. Fruit were then inoculated with *C. gloeosporioides* and decay was monitored for 6 days postinoculation (dpi). (**A**) Area of decay caused by *C. gloeosporioides*. (**B**) Representative picture of mango fruit treated with 8 mM Phe and control, 4 dpi with *C. gloeosporioides*. (**C**) Area under the *L. theobromae* disease progress curve (AUDPC) during 4 dpi. (**D**) Representative picture of mango fruit treated with 8 mM Phe compared to nontreated control, 4 dpi. Values are mean ± SE. Different letters indicate significant difference ($p \leq 0.05$) by one-way ANOVA.

Figure S2

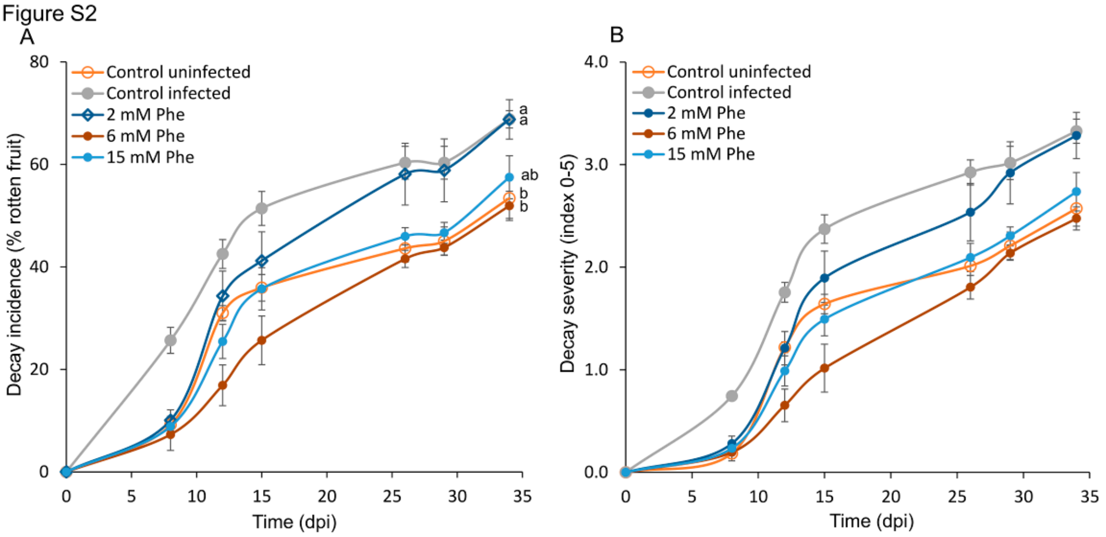

Figure S2. Effect of postharvest treatment with phenylalanine (Phe) on green mold decay caused by *P. digitatum* inoculation in mandarin fruit. 'Orr' mandarin fruit were treated with water or different concentrations (2, 6, 15 mM) of Phe, incubated at room temperature for 2 days. The fruit were then inoculated with *P. digitatum* and monitored for 35 days postinoculation (dpi). (**A**) Decay incidence (percentage of rotten fruit). (**B**) Decay severity (index 0–5). Values are mean ± SE. Different letters indicate significant difference ($p \leq 0.05$) by one-way ANOVA.

Figure S3

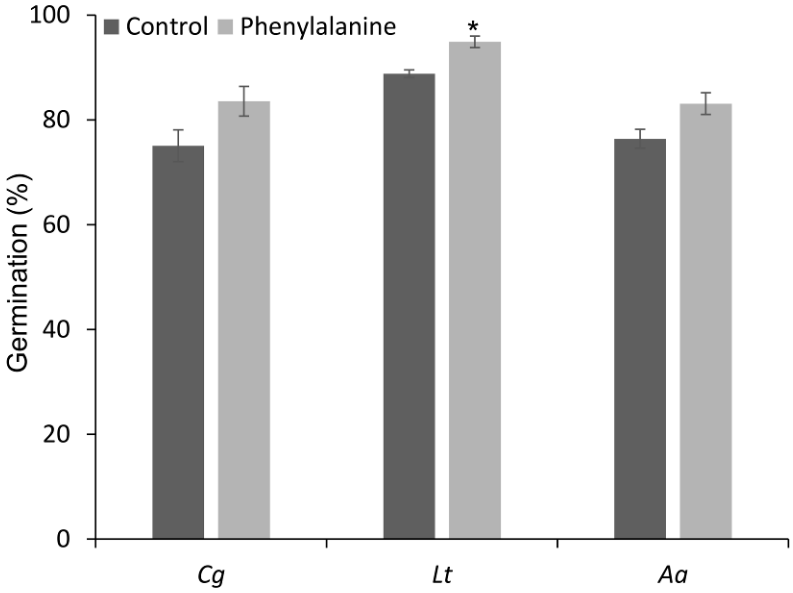

Figure S3. Direct effect of phenylalanine on conidial germination. Percentage of conidial germination of *C. gloeosporioides* (*Cg*), *L. theobromae* (*Lt*) or *A. alternata* (*Aa*) in the presence of 8 mM phenylalanine or water (control), counted after 20 h. Values are mean ± SE. Asterisk (*) represents a statistically significant difference between phenylalanine treatment and controls by t-test ($p \leq 0.05$).

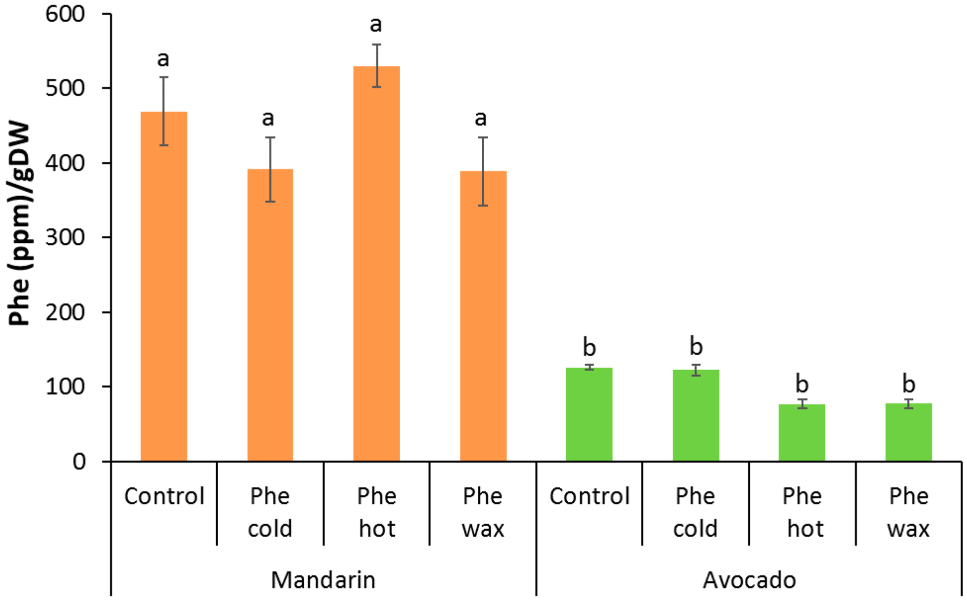

Figure S4. Phenylalanine residue in treated and untreated mandarin and avocado fruit. 'Michal' mandarin and 'Reed' avocado were dipped for 30 seconds in water (control) or in "cold" (20 °C) or hot (50 °C) 8 mM phenylalanine or embedded in polyethylene wax. Five hours later the phenylalanine concentration was evaluated.
